# Supplementary material for: Mortality and Major Cardiovascular Events among Patients with Multiple Myeloma: Analysis from a Nationwide French Medical Information Database
Source: Cancers (Basel). 2022 Jun 21;14(13):3049. doi: 10.3390/cancers14133049 (PMC9264957; doi:10.3390/cancers14133049)
Supplement: Supplementary file 1 [file cancers-14-03049-s001.zip › cancers-1761636-supplementary.pdf]

cancers MDPI

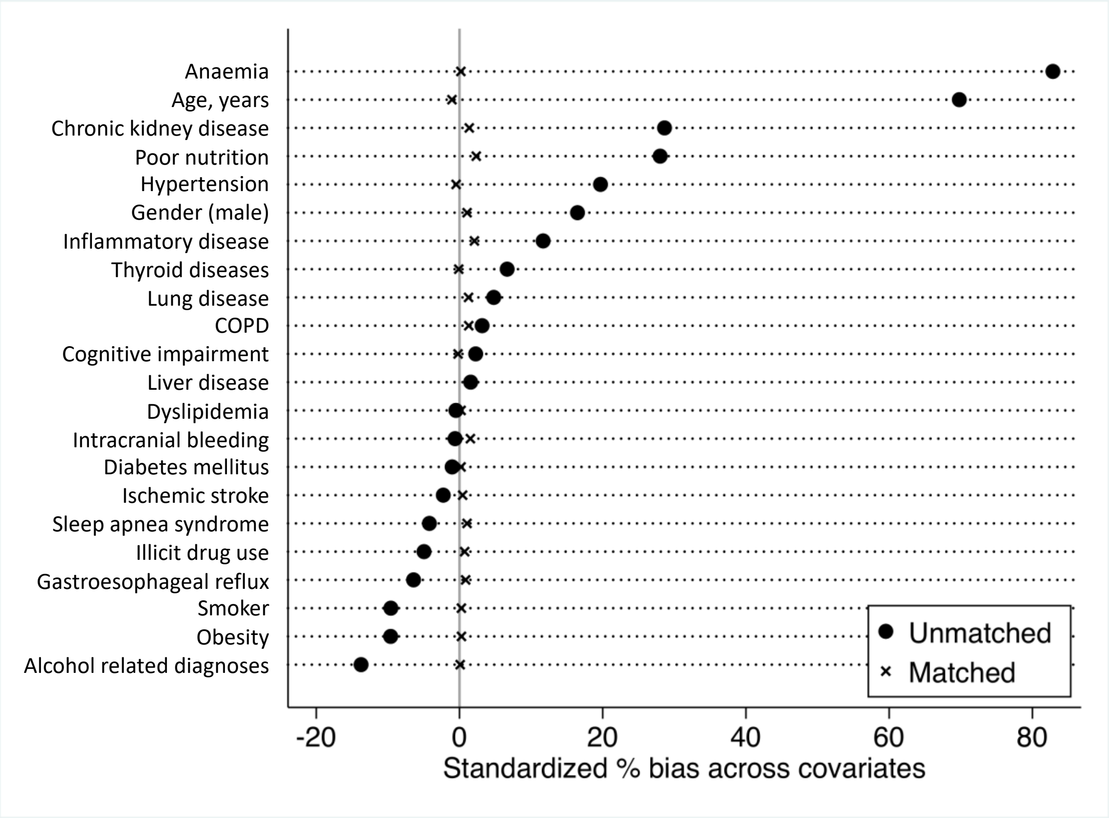

Figure S1. Standardized percentages of bias across main baseline characteristics in unmatched and matched patients with myeloma and no myeloma.

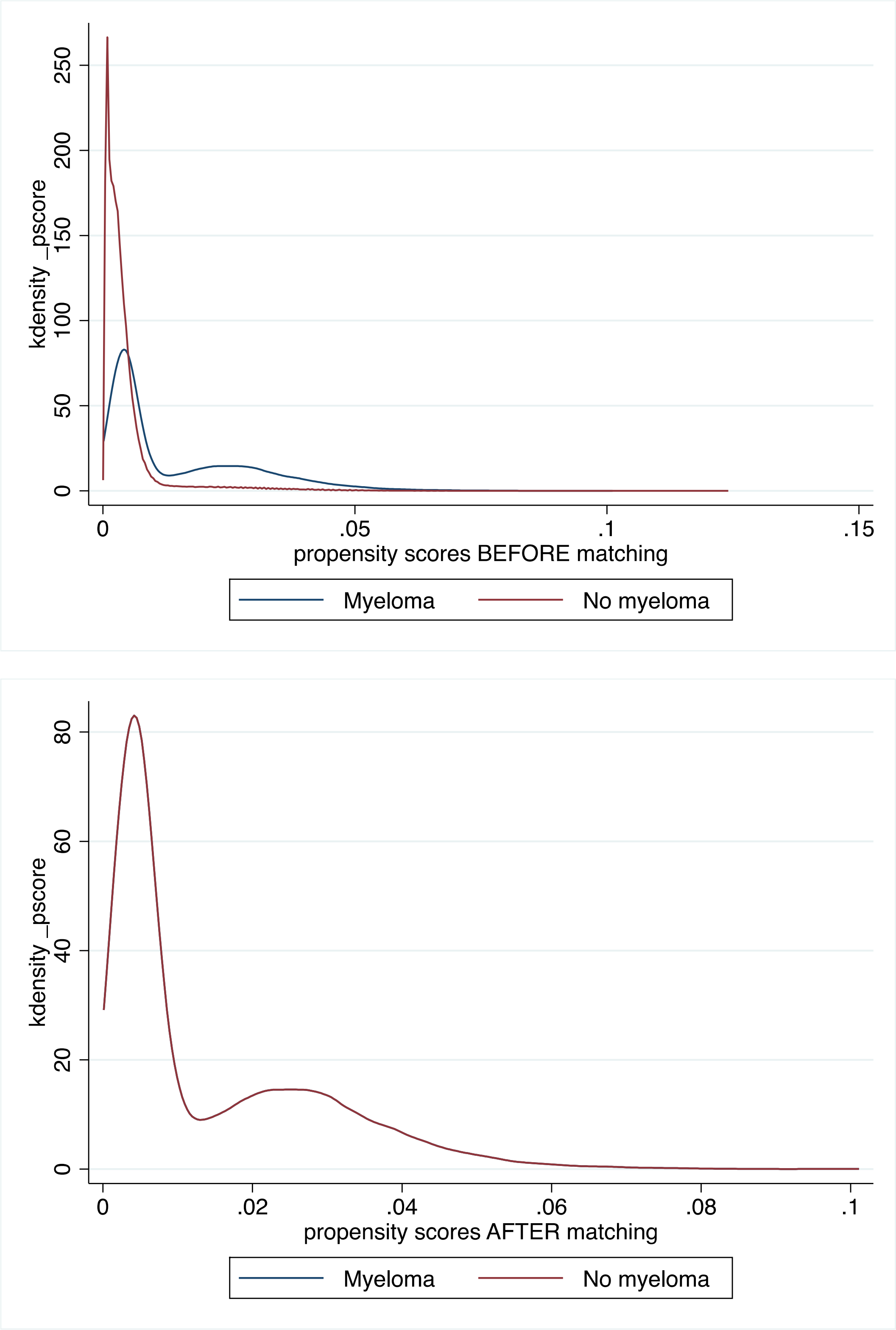

Figure S2. Propensity score distribution for unmatched and matched populations of patients with myeloma and no myeloma.

Table S1. Incident outcomes in the whole unmatched population*.

	Without Multiple Myeloma	(n=3,350,729)	With Multiple Myeloma	(n=15,774)	*p*
	**Number of events**	**Incidence, %/y (95% CI)**	**Number of events**	**Incidence, %/y (95% CI)**	
All-cause death	936,232	5·98 (5·97-6·00)	10,524	20·02 (19·65-20·41)	<0·0001
Cardiovascular death	191,168	1·22 (1·22-1·23)	1,053	2·00 (1·89-2·13)	<0·0001
Myocardial infarction	99,998	0·65 (0·64-0·65)	449	0·86 (0·79-0·95)	<0·0001
Ischaemic stroke	106,662	0·69 (0·68-0·69)	440	0·85 (0·77-0·93)	<0·0001
Major bleeding	236,234	1·55 (1·54-1·55)	1,784	3·61 (3·44-3·78)	<0·0001
Intracranial bleeding	77,203	0·50 (0·49-0·50)	539	1·03 (0·95-1·12)	<0·0001

*The mean follow-up was 4·7±1·8 years (median 5·4, IQR 5·0-5·8 years).

Table S2. Baseline characteristics of unmatched patients in the three previous months seen in French hospitals in 2013 with at least 5 years of follow-up.

	Without Multiple Myeloma (n=3,350,729)	With Multiple Myeloma (n=4537)	*p*	Total (n=3,355,266)
Age, years	59·1±21·5	71·7±11·8	<0·0001	59·2±21·5
Sex (male)	1568467 (46·8)	2481 (54·7)	<0·0001	1570948 (46·8)
Hypertension	1022172 (30·5)	1549 (34·1)	<0·0001	1023721 (30·5)
Diabetes mellitus	465033 (13·9)	531 (11·7)	<0·0001	465564 (13·9)
Heart failure	351359 (10·5)	568 (12·5)	<0·0001	351927 (10·5)
History of pulmonary oedema	25916 (0·8)	46 (1·0)	0·06	25962 (0·8)
Valve disease	120980 (3·6)	173 (3·8)	0·46	121153 (3·6)
Previous endocarditis	4486 (0·1)	12 (0·3)	0·02	4498 (0·1)
Dilated cardiomyopathy	77368 (2·3)	122 (2·7)	0·09	77490 (2·3)
Coronary artery disease	357923 (10·7)	386 (8·5)	<0·0001	358309 (10·7)
Previous myocardial infarction	57239 (1·7)	38 (0·8)	<0·0001	57277 (1·7)
Previous PCI	88528 (2·6)	65 (1·4)	<0·0001	88593 (2·6)
Previous CABG	12205 (0·4)	9 (0·2)	0·06	12214 (0·4)
Vascular disease	289114 (8·6)	287 (6·3)	<0·0001	289401 (8·6)
Atrial fibrillation	321479 (9·6)	499 (11·0)	0·001	321978 (9·6)
Previous pacemaker or ICD	104089 (3·1)	115 (2·5)	0·03	104204 (3·1)
Ischaemic stroke	63509 (1·9)	54 (1·2)	0·0005	63563 (1·9)
Intracranial bleeding	35056 (1·0)	41 (0·9)	0·35	35097 (1·0)
Smoker	231029 (6·9)	166 (3·7)	<0·0001	231195 (6·9)
Dyslipidaemia	441094 (13·2)	462 (10·2)	<0·0001	441556 (13·2)
Obesity	355793 (10·6)	279 (6·1)	<0·0001	356072 (10·6)
Alcohol-related diagnoses	187581 (5·6)	107 (2·4)	<0·0001	187688 (5·6)
Chronic kidney disease	117537 (3·5)	348 (7·7)	<0·0001	117885 (3·5)
Lung disease	340348 (10·2)	398 (8·8)	0·002	340746 (10·2)
Sleep apnoea syndrome	134202 (4·0)	109 (2·4)	<0·0001	134311 (4·0)
COPD	185911 (5·5)	199 (4·4)	0·001	186110 (5·5)
Liver disease	114867 (3·4)	128 (2·8)	0·02	114995 (3·4)
Thyroid diseases	182181 (5·4)	259 (5·7)	0·42	182440 (5·4)
Inflammatory disease	176442 (5·3)	185 (4·1)	0·0003	176627 (5·3)
Anaemia	272393 (8·1)	1118 (24·6)	<0·0001	273511 (8·2)
Previous cancer	486308 (14·5)	4537 (100·0)	<0·0001	490845 (14·6)
Poor nutrition	127872 (3·8)	292 (6·4)	<0·0001	128164 (3·8)
Cognitive impairment	114381 (3·4)	161 (3·5)	0·62	114542 (3·4)

Values are n (%) or mean ± SD. CABG = coronary artery bypass graft; COPD = chronic obstructive pulmonary disease; PCI = percutaneous coronary intervention; SD = standard deviation.

Table S3. Baseline characteristics of matched patients in the three previous months.

	Without Multiple Myeloma (n=4537)	With Multiple Myeloma (n=4537)	*p*	Total (n=9074)
Age, years	71·7±11·9	71·7±11·8	0·93	71·7±11·9
Sex (male)	2473 (54·5)	2481 (54·7)	0·87	4954 (54·6)
Hypertension	1557 (34·3)	1549 (34·1)	0·86	3106 (34·2)
Diabetes mellitus	525 (11·6)	531 (11·7)	0·84	1056 (11·6)
Heart failure	609 (13·4)	568 (12·5)	0·2	1177 (13·0)
History of pulmonary oedema	52 (1·1)	46 (1·0)	0·54	98 (1·1)
Valve disease	243 (5·4)	173 (3·8)	0·0004	416 (4·6)
Previous endocarditis	15 (0·3)	12 (0·3)	0·56	27 (0·3)
Dilated cardiomyopathy	123 (2·7)	122 (2·7)	0·95	245 (2·7)
Coronary artery disease	600 (13·2)	386 (8·5)	<0·0001	986 (10·9)
Previous myocardial infarction	105 (2·3)	38 (0·8)	<0·0001	143 (1·6)
Previous PCI	127 (2·8)	65 (1·4)	<0·0001	192 (2·1)
Previous CABG	24 (0·5)	9 (0·2)	0·01	33 (0·4)
Vascular disease	447 (9·9)	287 (6·3)	<0·0001	734 (8·1)
Atrial fibrillation	627 (13·8)	499 (11·0)	<0·0001	1126 (12·4)
Previous pacemaker or ICD	220 (4·8)	115 (2·5)	<0·0001	335 (3·7)
Ischaemic stroke	49 (1·1)	54 (1·2)	0·62	103 (1·1)
Intracranial bleeding	30 (0·7)	41 (0·9)	0·19	71 (0·8)
Smoker	161 (3·5)	166 (3·7)	0·78	327 (3·6)
Dyslipidaemia	474 (10·4)	462 (10·2)	0·68	936 (10·3)
Obesity	262 (5·8)	279 (6·1)	0·45	541 (6·0)
Alcohol-related diagnoses	96 (2·1)	107 (2·4)	0·43	203 (2·2)
Chronic kidney disease	334 (7·4)	348 (7·7)	0·58	682 (7·5)
Lung disease	404 (8·9)	398 (8·8)	0·82	802 (8·8)
Sleep apnoea syndrome	106 (2·3)	109 (2·4)	0·84	215 (2·4)
COPD	204 (4·5)	199 (4·4)	0·8	403 (4·4)
Liver disease	111 (2·4)	128 (2·8)	0·27	239 (2·6)
Thyroid diseases	270 (6·0)	259 (5·7)	0·62	529 (5·8)
Inflammatory disease	183 (4·0)	185 (4·1)	0·92	368 (4·1)
Anaemia	1107 (24·4)	1118 (24·6)	0·79	2225 (24·5)
Previous cancer	1017 (22·4)	4537 (100·0)	<0·0001	5554 (61·2)
Poor nutrition	282 (6·2)	292 (6·4)	0·67	574 (6·3)
Cognitive impairment	159 (3·5)	161 (3·5)	0·91	320 (3·5)
Illicit drug use	0 (0·0)	2 (0·0)	0·16	2 (0·0)

Values are n (%) or mean ± SD. CABG = coronary artery bypass graft; COPD = chronic obstructive pulmonary disease; PCI = percutaneous coronary intervention; SD = standard deviation.

Table S4. Incident outcomes (in the three previous months) in the unmatched population.

	Without Multiple Myeloma (n=3,350,729)		With Multiple Myeloma (n=4537)		*p*
	**Number of events**	**Incidence, %/y (95% CI)**	**Number of events**	**Incidence, %/y (95% CI)**	
All-cause death	936232	5·98 (5·97-6·00)	2840	17·55 (16·92-18·21)	<0·0001
Cardiovascular death	191168	1·22 (1·22-1·23)	285	1·76 (1·57-1·98)	<0·0001
Myocardial infarction	99998	0·65 (0·64-0·65)	132	0·82 (0·69-0·98)	0·11
Ischaemic stroke	106662	0·69 (0·68-0·69)	125	0·78 (0·65-0·93)	0·91
Major bleeding	236234	1·55 (1·54-1·55)	648	4·38 (4·06-4·74)	<0·0001
Intracranial bleeding	77203	0·50 (0·49-0·50)	138	0·86 (0·73-1·01)	<0·0001

Table S5. Incident outcomes (in the 3 previous months) in the matched population.

	Without Multiple Myeloma (n=4537)		With Multiple Myeloma (n=4537)		*p*
	**Number of events**	**Incidence, %/y (95% CI)**	**Number of events**	**Incidence, %/y (95% CI)**	
All-cause death	1843	9·57 (9·14-10·01)	2840	17·55 (16·92-18·21)	<0·0001
Cardiovascular death	338	1·75 (1·58-1·95)	285	1·76 (1·57-1·98)	0·48
Myocardial infarction	169	0·89 (0·76-1·03)	132	0·82 (0·69-0·98)	0·26
Ischaemic stroke	182	0·95 (0·83-1·10)	125	0·78 (0·65-0·93)	0·04
Major bleeding	397	2·13 (1·93-2·35)	648	4·38 (4·06-4·74)	<0·0001
Intracranial bleeding	144	0·75 (0·64-0·88)	138	0·86 (0·73-1·01)	0·13

Table S6. Hazard ratios associated with multiple myeloma in the three previous months (vs without) for incident outcomes.

	Model A	Model B	Model C	Model D
All-cause death	2·534 (2·442-2·629)	1·840 (1·773-1·909)	1·789 (1·724-1·856)	1·738 (1·639-1·843)
Cardiovascular death	1·237 (1·102-1·390)	0·881 (0·784-0·989)	0·926 (0·824-1·040)	0·936 (0·799-1·097) *
Myocardial infarction	1·149 (0·969-1·363)	0·857 (0·723-1·017)	0·924 (0·779-1·096)	0·887 (0·706-1·115) †
Ischaemic stroke	1·010 (0·848-1·204)	0·748 (0·627-0·891)	0·772 (0·648-0·920)	0·777 (0·618-0·976) ‡
Major bleeding	2·573 (2·382-2·780)	1·916 (1·774-2·070)	2·074 (1·920-2·240)	1·940 (1·711-2·199)
Intracranial bleeding	1·542 (1·305-1·823)	1·166 (0·986-1·377)	1·176 (0·995-1·390)	1·127 (0·891-1·424)

Model A: unadjusted. Model B: adjusted for age and sex. Model C: adjusted for all risk factors and non-cardiovascular comorbidities: age, sex, hypertension, smoker, dyslipidemic, diabetes mellitus, obesity, alcohol-related diagnoses, previous ischaemic stroke, intracranial bleeding, chronic kidney disease, lung disease, sleep apnoea syndrome, chronic obstructive pulmonary disease, liver disease, gastroesophageal reflux, thyroid diseases, inflammatory disease, anaemia, poor nutrition, cognitive impairment, and illicit drug use. Model D: propensity score matched analysis adjusted for variables mentioned for Model C.* Hazard ratio = 0·809 (0·691-0·948), p=0·009, by Fine and Gray model for competing risks of cardiovascular and non-cardiovascular death. † Hazard ratio = 0·743 (0·591-0·935), p<0·0001, by Fine and Gray model for competing risks of myocardial infarction and all-cause death. ‡ Hazard ratio = 0·647 (0·515-0·812), p=0·009, by Fine and Gray model for competing risks of ischaemic stroke and all-cause death.
